# Morphological variation in *Cynodon dactylon* (L.) Pers., and its relationship with the environment along a longitudinal gradient

**DOI:** 10.1186/s41065-020-00117-1

**Published:** 2020-02-12

**Authors:** Miaoli Wang, Jingxue Zhang, Zhipeng Guo, Yongzhuo Guan, Gen Qu, Jianyu Liu, Yuxia Guo, Xuebing Yan

**Affiliations:** 1 College of Animal & Veterinary Science, Henan Agricultural University, Zhengzhou, 450002 China; 2 College of Animal Science and Technology, Yangzhou University, Yangzhou, 225000 China

**Keywords:** *Cynodon dactylon*, Wild beomudagrass, Longitude, Phenotypic plasticity, Soil nutrients, Climate

## Abstract

**Background:**

Geographical variation in morphological traits may reflect evolutionary patterns of morphological adaptability along environmental gradients. Comprehensive information on longitudinal patterns of morphological trait variation is very meaningful to explore morphological diversity and evolutionary trends in widespread bermudagrass.

**Methods:**

To explore the spatial patterns of morphological traits, we investigated 10 morphological traits of bermudagrass and 10 soil nutrient indexes and collected local climate data for 13 different regions from 119°E to 105°E along the latitude 34°N.

**Results:**

Considerable variations in morphological traits were observed at different longitudes, and the variations in most of the evaluated traits within populations were lower than those among populations. All of the 13 different longitudinal sites were divided into three groups based on morphological traits by cluster analysis. The major sources of diversity at the different longitudes were leaf length of the erect shoot, leaf width of the erect shoot, and the internode lengths of the erect shoot and stolon as determined by principal component analysis. Pearson correlation analysis also indicated that longitude was significantly and negatively correlated with these traits as well. Mean average rainfall was significantly correlated with leaf length of the erect shoot and the internode lengths of the erect shoot and stolon, while mean average temperature was only significantly correlated with internode length of the erect shoots. Available sulfur was significantly correlated with internode length of the erect shoot, plant height, and reproductive branch height, while the exchangeable Ca was significantly correlated with internode lengths of the erect shoot and stolon. Soil pH was significantly correlated with the internode length of the stolon. Longitude is an important factor that affects morphological trait variation in wild bermudagrass, and the leaves of the erect shoot and the internode length enlarged significantly with the collection sites moving from east to west.

**Conclusion:**

Different combinations and interactions of environmental factors (soil and climate) along a longitudinal gradient may have strong effects on one or more morphological traits of bermudagrass.

## Background

Phenotypic plasticity is a widespread and important phenomenon that allows plants to adapt to the environment, and the adaptive mechanism may be related to the specific environments in which the plants live; that is, different environments may select for plants that have different phenotypic plasticity mechanisms [1–4]. It is generally accepted that fluctuating environmental conditions play a key role in the morphological variation in plants [5, 6]. Morphological variation in plants is generally a feedback for the changing climatic conditions and reflects the adaptive evolution [7–12], while the soil factor has apparently shown to be a driving force to determine the morphological traits of plants [13–16]. Morphological variation can evolve in response to environmental variability and may show clear patterns in geographical regions; for example, the distribution of *Drosophila* species is implicated in its innate thermal tolerance limits, and a majority of measured morphological traits of *Sorghum bicolor* Moench show significant regional differences, etc. [17–20]. However, few studies have addressed whether plant species would show appreciable changes or a significant pattern of morphology along a longitudinal gradient. The climatic variability hypothesis suggests that individuals of a species may exhibit larger morphological variation with increasing climatic variability, which could happen in areas of lower precipitation at decreased longitude [21, 22]. The leaf length and width of *Pistacia atlantica* Desf. subsp. *Atlantica* were found to be significantly related to precipitation, and precipitation is the major factor that determines plant functional traits in *Stipa* species [23, 24]. It is generally accepted that precipitation varies considerably with changes in longitude in many areas of the world. In the climate of China, precipitation also decreases linearly with increasing longitude from east to west in regions far from the sea [25]. At present, there is no information about the effects of climatic changes on morphological traits in widespread plant species, which makes it unclear how plants adapt to regular changes in climate, especially precipitation.

Bermudagrass, *Cynodon dactylon* (L.) Pers., is an important perennial grass species in the botanical family Poaceae [26]. Originally native to Africa, *C. dactylon* has become naturalized in many places around the world and is widely distributed in tropical, subtropical, and temperate coastal areas [27]. *C. dactylon* has the advantages of strong viability, rapid reproduction, and resistance to trampling, making it a suitable grass for lawns, livestock forage, soil conservation, and remediation of contaminated soils [12, 13]. As early as the 1960s, researchers at Oklahoma State University divided *C. dactylon* germplasm into six variants based on their growth habits, leaf color, plant type, and somatic chromosome count [14]. Bermudagrass was also divided into four biological types based on external morphological traits including leaf color, stem color, and shaped type in Mauritius [28]. Harlan et al. (1970) divided the *C. dactylon* collections from around the world into nine species and 10 variants, and Taliaferro reviewed and revised the taxonomy of *C. dactylon* [14, 29]. In the previous studies that explored the geographical clines in morphology for *C. dactylon*, most were focused on different habitats [30]. We can ask two questions; (1) how does the widespread *C. dactylon* adapt to the environmental changes along a longitudinal cline by morphological variation? And (2) what relationships are there between morphological traits and climate? At present, no studies have been conducted on morphological variation in *C. dactylon* that are strictly related to different longitudes to answer these questions. Thus, in our study, we measured 10 quantitative morphological traits in plants of *C. dactylon* collected from locations at 13 different longitudes and analyzed the relationships between these variations and the local soil and climatic conditions. The objectives of this research were to i) analyze the variability in 10 quantitative morphological traits of *C. dactylon* collected at sites representing 13 different longitudes, ii) explore the pattern of morphological variation among and within populations, and iii) evaluate the relationships between morphological traits, soil, and climate at the different longitudes.

## Material and methods

### Plant material

In August 2016, a total of 260 wild bermudagrass individuals were sampled as representatives of 13 populations growing in regions at different longitudes from 119°E to 105°E along latitude 34°N in southern China (Table 1). Meteorological data, including the mean average rainfall (MAR) and the mean average temperatures (MAT) over thirty years (1981–2010) were recorded for the 13 sampled regions (Table 1).

**Table 1:** Geographical and climatic factors of bermudagrass in 13 different longitude regions

Code	Region	Habitat	Longitude (E)	Latitude (N)	Altitude (m)	MAT (°C)	MAR (mm)
1	Lianyungang	Island	119°27′06″	34°46′09″	50	14.5	883.9
2	Tancheng	Roadside	118°16′08″	34°38′37″	30	13.8	832.9
3	Zaozhuang	Roadside	117°49′20″	34°38′48″	89	14.4	820.3
4	Shanxian	Roadside	116°09′11″	34°46′31″	30	14.2	621.4
5	Lankao	Roadside	114°44′55″	34°49′32″	60	14.3	631.1
6	Zhengzhou	Roadside	113°38′20″	34°54′04″	90	14.7	640.8
7	Luoyang	Roadside	112°19′30″	34°43′20″	210	14.4	637.2
8	Sanmenxia	Roadside	111°03′49″	34°42′29″	340	14.0	558.1
9	Tongguan	Roadside	110°13′18″	34°33′41″	540	13.1	602.9
10	Jingyang	Roadside	108°50′07″	34°32′32″	410	13.5	504.1
11	Fufeng	Roadside	107°52′41″	34°20′35″	570	12.8	569.9
12	Baoji	Ditch side	107°41′03″	34°21′54″	630	13.5	645.9
13	Tianshui	Roadside	105°57′34″	34°32′43″	1050	11.4	500.7

*MAT* mean average rainfall; *MAR* mean average temperature

### Morphological trait data collection

The morphological characters, including leaf length of the erect shoot (LLES), leaf width of the erect shoot (LWES), internode length of the erect shoot (ILES), diameter of the erect shoot (DES), leaf length of the stolon (LLS), leaf width of the stolon (LWS), internode length of the stolon (ILS), diameter of the stolon (DS), plant height (PHT), and reproductive branch height (RBH) were measured for 20 individual plants per site. The sampling method was as described in our previous study [31]. The samples were measured with the aid of a caliper during the highest growth period in August. The fourth mature leaf from the top of the erect shoot to the base was selected for measurement of LLES and LWES, and the fourth section stems from the top of the erect shoot to the basal part were used for measurement of DES and ILES. The methods used to measure the stolon characters LLS, LWS, DS, and ILS were identical to those used to measure the erect shoot above. The natural height of bermudagrass was measured for PHT, and the vertical height from the surface to the natural top of the reproductive branch was measured for RBH. The values for 10 quantitative morphological traits were used for further data analysis. The climate data for the sampling sites was obtained from the website of the China meteorological data network.

### Soil chemical characteristics

The soil samples (0–20 cm depth) were collected separately under the cover of the wild bermudagrass from 20 corresponding quadrats (10 m × 10 m) at each site, and the soil was collected using the principle of multi-point sampling. The soil samples were taken within different quadrats and mixed at each site. The samples were then sealed in polyethylene bags using the quarterly method to divide the soil into portions of approximately 1 kg. In order to facilitate the different analyses that require grinding and screening, the collected soil samples were transported to the laboratory and allowed to air-dry at 25 °C. The soil samples were analyzed for total nitrogen (TN), available nitrogen (AN), available sulfur (AS), available kalium (AK), available phosphate (AP), soil organic matter (SOM), exchangeable Ca, exchangeable Na, exchangeable Mg, and pH based on standard methodologies [32]. Briefly, the SOM was determined by the potassium dichromate wet combustion procedure. TN and AN were analyzed using the Kjeldahl method and the alkaline nitrogen diffusion method, respectively. The AS, AK, AP were determined using the barium chloride turbidimetric method, flame emission spectrometry and Olsen method, respectively. The exchangeable Ca, exchangeable Na, and exchangeable Mg were measured by continuum-source atomic-absorption spectrometry. Soil pH was tested with a glass electrode (soil to water ratio = 1:2.5).

### Statistical analysis

The statistical parameters including maximum, minimum, mean, standard deviation (SD), and coefficient of variation (CV) for each trait at each longitude site were estimated by using EXCEL. Nested variation analysis was used to calculate the significance among populations and within populations for 10 morphological traits of wild bermudagrass, which were collected from 13 different sites. Within-population and among-population variance components were calculated to determine the percentage of variability owing to the longitude and the local micro-environmental changes (SPSS 22.0). We explored the relationships between morphological traits, longitude, soil, and climate by Pearson’s correlation analysis and presented resulted by HemI 1.0.3.3 software. The 13 different longitude sites were clustered based on the 10 morphological traits using cluster analysis (CA). Principle component analysis (PCA) of the 10 morphological traits was used to evaluate the diversity of morphological traits in bermudagrass. Figures were drawn with Sigmaplot 10.0 (Systat Software Inc.) and the R package.

## Results

Descriptive statistics of morphological traits and soil nutrients along a longitudinal gradient.

The maximum, minimum, mean, SD, and CV of 10 morphological traits at each site are given in Table 2. The CVs for the 10 traits ranged from 13.78% (LWES) to 34.13% (LLES). The CV values for LLES, ILES, LLS, ILS, PHT, and RBH were > 20%, meaning that there were larger morphological variations in those traits along the longitudinal gradient. The CV values for LWES, DES, LWS, and DS were < 20% but > 10%, which means that there were smaller morphological variations in these traits along the longitudinal gradient, and the CV values for the length and height characters were greater than for the width characters. Observable morphological variation existed in bermudagrass along the longitudinal gradient, and the 10 quantitative traits showed moderate and higher morphological variation.

**Table 2:** Average values for morphological traits of bermudagrass germplasm collected at 13 different longitudinal sites along latitude 34^o^N

Code	LLES (mm)	LWES (mm)	ILES (mm)	DES (mm)	LLS (mm)	LWS (mm)	ILS (mm)	DS (mm)	PHT (cm)	RBH (cm)
1	53.48	2.81	23.76	0.99	73.08	3.23	35.29	1.00	18.93	19.51
2	43.34	2.44	20.92	0.74	50.58	2.66	34.24	0.94	17.20	16.37
3	40.56	2.25	20.89	0.68	47.81	2.60	26.48	0.80	16.25	16.20
4	49.11	1.94	21.77	0.60	39.75	2.21	40.10	0.77	16.30	15.25
5	61.34	2.66	40.10	0.75	95.15	2.95	47.00	0.86	38.05	31.04
6	67.51	2.77	36.04	0.95	74.63	3.03	51.17	1.16	19.48	23.90
7	42.49	2.49	28.30	0.78	48.18	2.60	45.53	0.83	18.74	17.73
8	51.49	3.42	35.74	1.11	40.19	3.44	67.31	1.43	21.90	19.56
9	41.60	2.32	31.62	0.75	33.95	2.46	39.65	0.81	16.35	16.14
10	67.83	2.83	36.71	0.76	74.56	3.35	66.22	0.96	22.45	20.09
11	64.52	3.11	33.11	0.84	57.16	3.13	54.17	1.15	20.80	17.20
12	66.54	3.18	32.29	0.89	85.19	3.43	52.80	0.98	16.15	22.11
13	81.07	3.40	51.69	0.94	95.64	2.66	68.62	1.16	26.00	27.93
Maximum	81.07	3.42	51.69	1.11	95.64	3.44	68.62	1.43	38.50	31.04
Minimum	40.56	1.94	20.89	0.60	33.95	2.21	26.48	0.77	16.15	15.25
Mean	56.22	2.74	31.76	0.83	62.76	2.90	48.35	0.99	20.66	20.23
SD	12.83	0.45	8.85	0.14	21.42	0.40	13.39	0.19	6.01	4.85
CV (%)	22.82	16.52	27.85	16.98	34.13	13.78	27.70	19.37	29.08	23.97

*LLES* leaf length of the erect shoot; *LWES*leaf width of the erect shoot; *ILES* internode length of the erect shoot; *DES* diameter of the erect shoot; *LLS* leaf length of the stolon; *LWS* leaf width of the stolon; *ILS* internode length of the stolon; *DS* diameter of the stolon; *PHT* plant height; *RBH* reproduction branch height

The mean values for the soil nutrient contents at the different longitude sites are shown in Table 3. There was no obvious distribution pattern for soil nutrients along the longitudinal gradient. The 0soil nutrient CVs were very high, and all indexes were > 27% along the longitudinal gradient. The CV values were in the following relative order: AP > exchangeable Mg > AS>exchangeable Na > AK > exchangeable Ca > SOM > TN > AN>pH. In addition, the variation in pH had almost no effect compared to the magnitude of the environmental changes along the longitudinal gradient.

**Table 3:** Soil nutrient contents and pH at 13 collection sites for wild bermudagrass at different longitudes along latitude 34^o^N

Code	TN (g∙kg^−1^)	AN (mg∙kg^−1^)	SOM (g∙kg^−1^)	AK (mg∙kg^−1^)	AS (mg∙kg^−1^)	AP (mg∙kg^−1^)	Exchangeable Ca (g∙kg^−1^)	Exchangeable Na (g∙kg^−1^)	Exchangeable Mg (g∙kg^−1^)	pH
1	0.87	110.50	18.70	122.00	2.02	12.30	0.90	0.12	0.13	6.72
2	1.35	127.00	32.35	285.00	3.88	68.95	2.80	0.13	0.25	6.87
3	1.26	102.50	28.30	203.00	2.81	17.25	2.59	0.08	0.42	6.87
4	1.50	120.00	33.85	243.00	3.38	25.30	6.11	0.08	0.26	6.98
5	1.07	83.50	23.10	265.00	8.13	26.00	5.81	0.32	0.54	7.39
6	1.11	91.50	17.00	329.00	3.85	78.10	4.46	0.20	0.36	6.75
7	2.09	101.00	40.25	490.00	6.60	31.40	8.30	0.09	0.23	7.37
8	1.09	89.00	25.85	476.00	4.61	23.70	8.89	0.12	0.33	7.47
9	1.17	109.50	21.95	293.00	3.23	56.60	7.73	0.10	0.14	7.50
10	0.89	71.00	24.00	743.00	3.30	42.50	8.63	0.23	0.76	7.87
11	0.90	169.50	15.60	215.50	3.23	34.00	9.53	0.09	0.24	7.69
12	2.04	93.00	46.10	481.00	2.95	68.70	8.30	0.15	0.37	6.87
13	1.16	170.00	31.95	199.50	9.73	19.40	8.96	0.27	0.25	7.21
Maximum	2.09	170.00	46.10	743.00	9.73	78.10	9.53	0.32	0.76	7.87
Minimum	0.87	71.00	15.60	122.00	2.02	12.30	0.90	0.08	0.13	6.72
Mean	1.27	110.62	27.62	334.23	4.44	38.78	6.39	0.15	0.33	7.20
SD	0.40	30.22	9.07	169.50	2.29	22.12	2.86	0.08	0.17	0.38
CV(%)	31.24	27.32	32.84	50.71	51.62	57.04	44.85	51.50	51.99	5.27

*TN* total nitrogen; *AN* available nitrogen; *SOM* soil organic matter; *AK* available potassium; *AS* available sulfur; *AP* available phosphate

Variation in morphological traits among and within populations.

The term “population” means that all individuals of the same species occupy a certain space in a certain period of time. We considered the samples collected in each site as representative of 13 populations in this study. High and significant levels of variation were found among and within populations for most measured traits (*P* < 0.01), and only LWS and DS showed no significant differences within populations (Table 4). The values of the variance components for all morphological traits among populations were > 15% and the range of changes was from 15.66% (LLES) to 51.53% (PHT). The morphological trait variation was mainly controlled by longitude and environmental factors among the populations. LWS had the lowest variance component value (5.26%), while DS had the highest variance component (49.72%) within populations. The variance components of the other traits ranged from 10 to 23% within populations, which indicated that the traits with lower variance component values within populations showed less genetic variation. Results from nested variation analysis showed that the variance components of morphological traits among individuals within populations were generally lower than those among populations.

**Table 4:** ANOVA for 10 morphological traits among and within 13 populations of wild bermudagrass

	Mean square (df)			F		Variance component (%)	
Traits	Populations ^a^	Ind (pop) ^b^	Error	Populations ^a^	Ind (pop) ^b^	Populations ^a^	Ind (pop) ^b^
LLES	3424.66	747.60	541.18	5.77**	1.38*	15.66	14.06
LWES	4.04	0.32	0.22	18.83**	1.49*	41.30	10.87
ILES	1638.69	379.50	0.05	6.60**	1.53**	16.69	17.85
DES	0.41	0.82	637.54	7.88**	1.60**	19.51	17.08
LLS	9016.14	983.78	0.25	14.14**	1.54**	33.48	13.75
LWS	3.98	0.29	353.60	16.08**	1.17	40.35	5.26
ILS	3931.52	586.11	353.60	11.12**	1.66**	26.22	15.75
DS	0.70	0.08	0.06	11.61**	1.31	17.13	49.72
PHT	743.18	43.10	21.72	34.21**	1.98**	51.53	16.11
RBH	419.40	97.86	56.12	7.47**	1.74**	17.33	22.49

Note: *, **: significant differences at probabilities of 0.05 and 0.01, respectively

Populations ^a^ stands for among populations; Ind (pop) ^b^ stands for within populations

### Correlation analysis

**Fig. 1:**
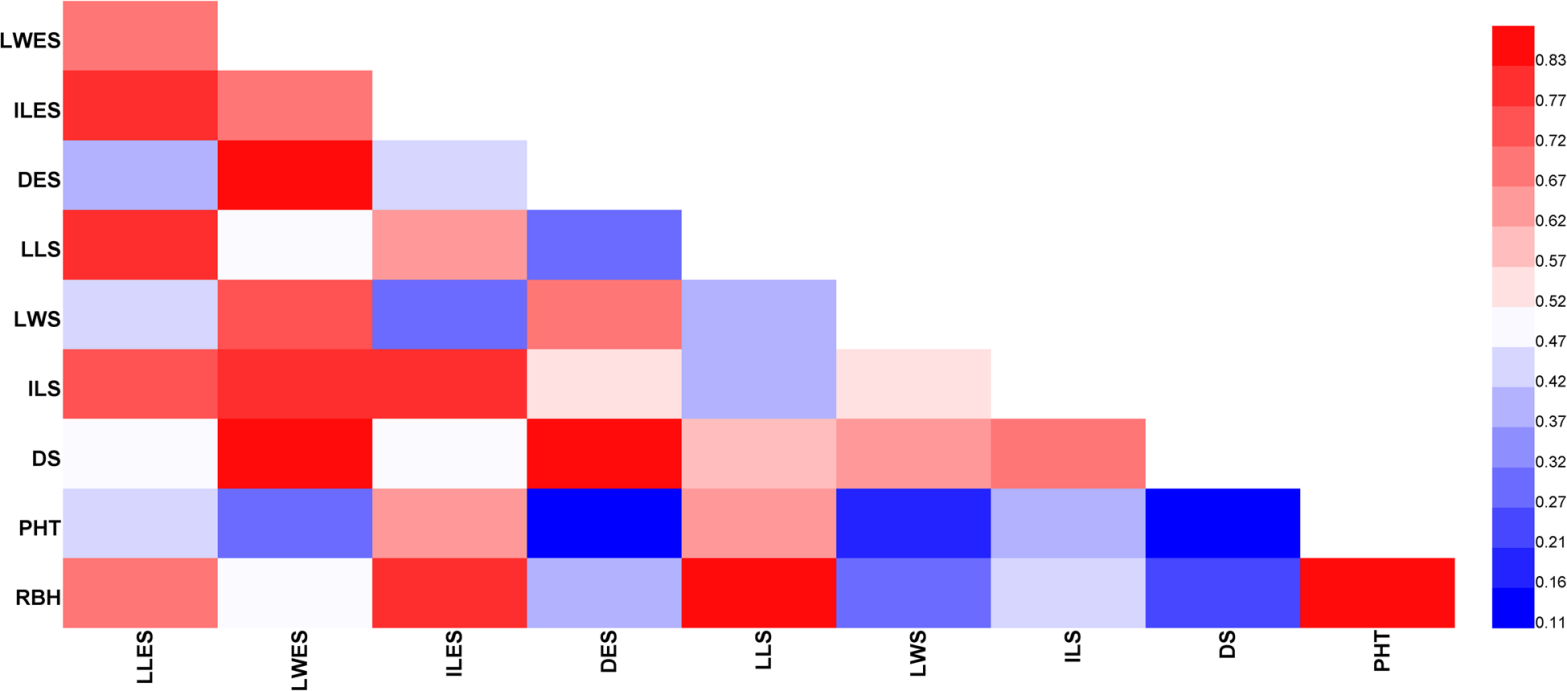
Heat map for the correlation coefficients between morphological traits of bermudagrass

**Fig. 2:**
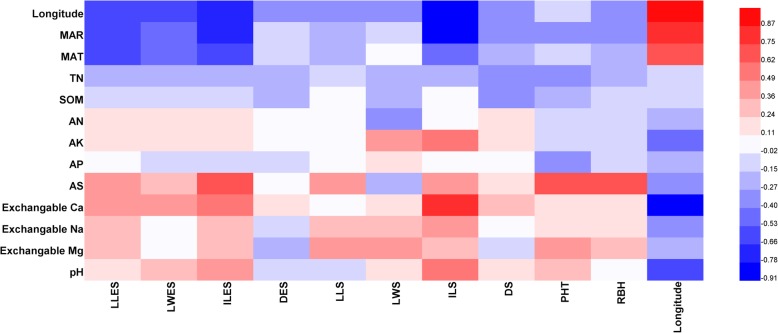
Heat map for the correlation of the soil nutrients, longitude, climate and morphological traits

There were significant correlations between the tested traits (Fig. 1). The results showed that leaf width traits were positively and significantly correlated with diameter traits in most cases. LLES and ILES were significantly correlated with LLS and ILS, separately. In addition, the height traits (PHT and RBH) showed positive and significant correlations with ILES and LLS. The results of correlation analyses between longitude and 10 morphological traits of bermudagrass showed that there were significant correlations for some morphological traits such as LLES (*P* < 0.05), LWES (*P* < 0.05), ILES (*P* < 0.01), and ILS (*P* < 0.01), and all tested traits were negatively correlated with longitude (Fig. 2). Thus, the larger morphological traits were associated with lower longitudes; the leaves on the erect shoots were wider, and internodes were longer in bermudagrass with decreasing longitude. LLES (*P* < 0.05), ILES (*P* < 0.01), and ILS (*P* < 0.01) were significantly and negatively correlated with MAR, and ILES (*P* < 0.05) was significantly and negatively correlated with MAT, which implied that bermudagrass has longer leaves and internodes in areas with colder temperatures with less rainfall.

ILES (*P* < 0.05) and ILS (*P* < 0.01) displayed positive and significant correlations with exchangeable Ca, and ILS (*P* < 0.05) was significantly and positively correlated with pH, which indicates that longer internodes are positively associated with higher contents of exchangeable Ca and higher pH. ILES (*P* < 0.01), PHT (*P* < 0.01), and RBH (*P* < 0.01) were significantly and negatively correlated with AS, which shows that plants of bermudagrass were taller with increasing AS content. In addition, the leaf width of the stolon and stolen diameter did not show significant correlations with any geo-climatic or soil parameters.

### Cluster analysis and principal component analysis of morphological traits

**Fig. 3:**
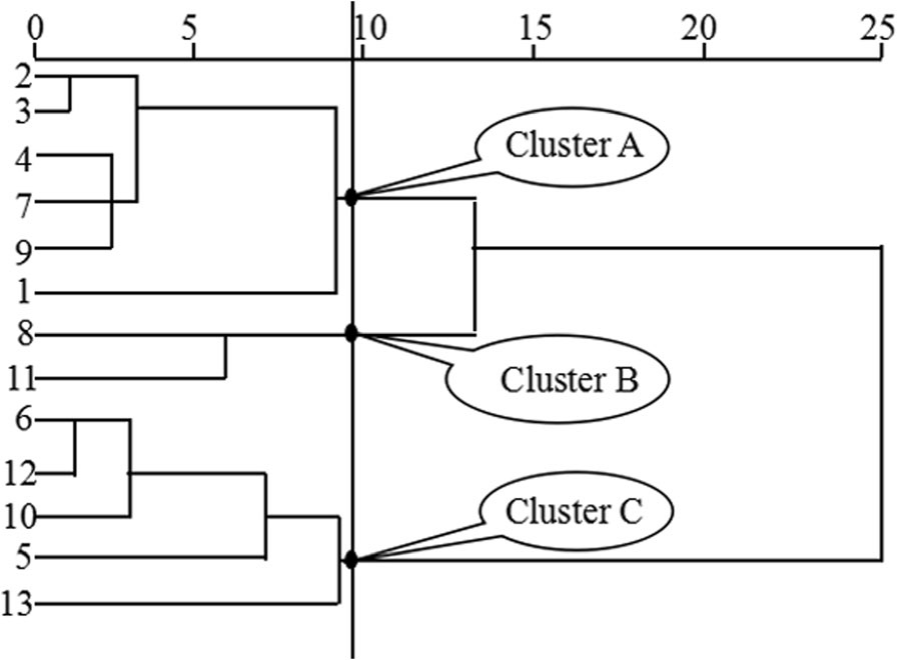
Cluster analysis of morphological traits

Figure 3 shows a dendrogram based on the cluster analysis of the morphological distance matrix for the 13 different longitudes. These different regions were divided into three clusters: the morphological traits included in clusters A, B, and C were in accordance with the approximate longitudes. Also, the bermudagrass populations were divided into many sub-populations by the complex environmental and geographical factors, which were observed at both low and high longitudes. Sites 1, 2, 3, 4, 7, and 9 grouped together in cluster A. This cluster contained plants with the minimum values of the morphological traits; plants were short with and narrow leaves and stems, except for plant height, which belonged to the dwarf and thin type. Sites 8 and 11 were included in cluster B. The bermudagrass plants in cluster B differed from the others by their maximum leaf width and diameter characteristics, which had the best creeping characters and belonged to the type of middle and wide. Cluster C consists of populations 5, 6, 10, 12, and 13 that had best upright characters and belonged to the tall and stocky plant type, because the plants in Cluster C had the maximum values for LLES, LLES, ILES, ILS, PHT, and RBH.

**Fig. 4:**
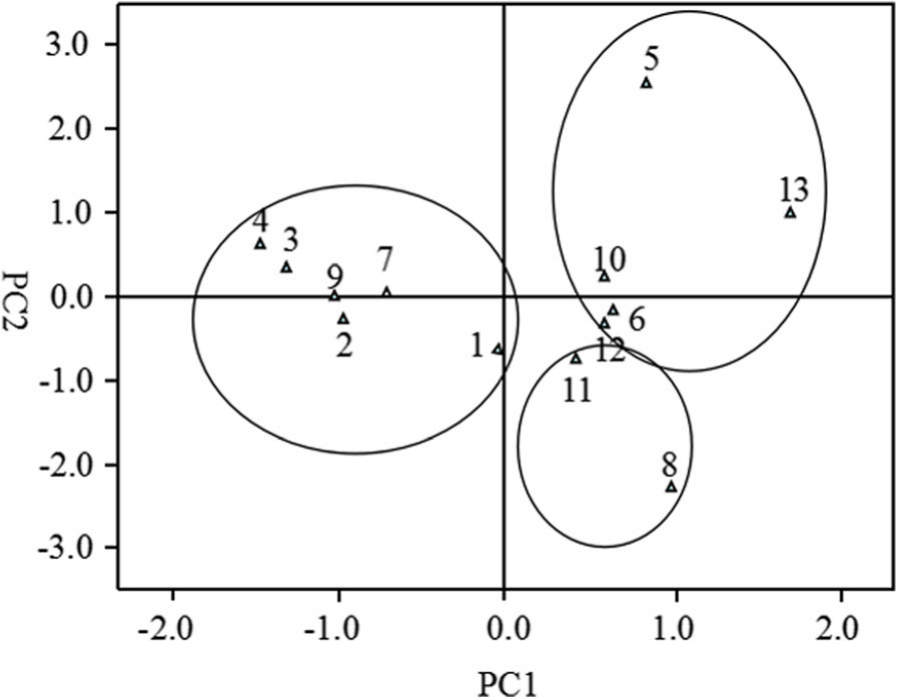
The distribution of bermudagrass populations on the PC1 and PC2 by using principal component

To estimate the relative significance of the evaluated traits and the longitudinal variation, we performed principal component analysis based on the 10 morphological traits. The PCA results showed that the first two PC axes (PC1 and PC2) explained 80.5% of the total multivariate variation (Table 5). The relatively large load in PC1 are LWES, ILES, LLES and ILS, and when the relatively large load in PC2 were PHT, DS, DES and RBH. The contribution of PC1 (60.03%) was much greater than that of PC2 (20.47%). Along the longitude gradient, morphological diversity in bermudagrass was mainly due to LWES, ILES, LLES, and ILS in PC1. In order to investigate the relationships between longitude and morphological traits and to determine the morphological variability, we calculated the Euclidean distances. The distribution of sites along the first two principle component axes partition similar groups, which confirmed the results of the cluster analysis (Fig. 4).

**Table 5:** Component matrix for principal component analysis (PCA) of the 10 morphological traits in bermudagrass

Traits	Principal components	
	PC1	PC2
LLES	0.862	0.210
LWES	0.902	−0.353
ILES	0.868	0.259
DES	0.715	−0.568
LLS	0.736	0.507
LWS	0.672	−0.424
ILS	0.835	−0.154
DS	0.731	−0.592
PHT	0.592	0.608
RBH	0.778	0.556

Note: PC1 explained 60.03% of the variance, and PC2 explained 20.47% of the variance

## Discussion

### The relationships between morphological traits and longitude

Previous studies have shown that morphological variations are significantly affected by geographical locations [33–37]. Morphological variation as a response to environmental changes at different geographic positions is common in *C. dactylon* [38, 39]. A study of 260 accessions of bermudagrass germplasm indicated that there is extensive morphological variation in *C. dactylon* populations along longitudinal gradients, which has been found in some other studies [31, 40, 41]. The correlation analysis showed that the traits of width and dimeter were seldom significantly correlated with the longitude, soil nuriture or climate. It seems these traits may be less susceptible to environmental changes and suggests that the mechanism for stress tolerance is found in bermudagrass, thereby reducing the change of irreversibly responding to varying environmental factors. In the contrast, most of other traits were significant correlated with each other and also significantly related with enviroment factors. These traits were significantly effected by environment factors including longitude variation so that they had high values in CV along the changes of longtitude. Leaf traits of the erect shoot and internode length were significantly affected by longitude, and these traits were also the main source of diversity in bermudagrass populations along a longitudinal gradient, which shows that longitude is an important factor that influences morphological variation in bermudagrass. In general, we observed a trend in which the morphological traits increased in size with decreasing longitude, suggesting that the larger sizes are an adaptation to drought, lower temperatures, and shorter growing seasons as well as longer and deeper periods of dormancy, enabling the plants to survive adverse conditions and to increase their competitive abilities. In many cases, plants are generally smaller and show reduced leaf trait sizes in order to reduce plant transpiration and maintain efficient utilization of the limited water resources under drought conditions [42–44]. Bermudagrass is a widespread species that is adaptable to adverse environments that shows a contrary strategy in morphological traits to adapt to dry and low temperature conditions. However, high temperatures did not cause increases in plant size along a latitudinal gradient in another study [31].

We have identified significant variation in morphological traits in bermudagrass among and within populations, which indicates that the different environments over a large scale had a remarkable influence on the morphological traits (Table 4). Variation in components of morphological traits within populations was lower than that among general populations, which means that longitude is the major factor influencing morphological variation in bermudagrass. Some studies have shown that genetic variation is the main determinant for adaptability of a species within and between populations [45–48]. Morphological variation is commonly influenced by genetic variation, environmental variation, or the interactions between them [4, 49]. Morphological variation in bermudagrass is mainly determined by the influence of longitude in this study, which can enhance the spreading to a large scale likewise.

### Effect of climatic and soil nutrients on morphological traits in bermudagrass

Previous studies in species such as rice and *Sarracenia purpurea* (pitcher plant) have investigated the spatial patterns of morphological trait variation along geographical gradients, and showed that the geographical patterns are shaped by edaphic and climatic factors [50, 51]. Morphological variations are well documented in many plant species such as *Afzelia africana* (African mahogany) and black plum, which are affected by climatic conditions [52–54]. Fruits and leaflets of *A. africana* show significant morphological variation under different climatic conditions [52]. Bejiga et al. (1996) discovered that climatic factors are significantly correlated with seed weight [55]. In our study, longer internodes were found in those areas with lower MAR and MAT. Previous studies showed that internode length in bermudagrass is significantly and negatively related to rainfall and temperature [31]. In areas with low rainfall and low temperatures, one adaptation mechanism for drought could be an increase in the internode length that would allow the leaf to more effectively absorb resources.

Soil nutrient factors are the most important components of the soil ecosystem and are necessary elements for plant growth [56, 57]. Soil nutrients are affected by many factors, such as climate, topography, microorganisms, and artificial factors [58, 59]. Both exchangeable Ca and pH showed significant variation along the longitudinal gradient, but most soil nutrients did not show an obvious difference. Bermudagrass morphological traits such as height and internode length were significantly affected by AS, exchangeable Ca, and pH in our study.

The combinations of and interactions between environmental factors (soil and climate) and longitude may significantly influence morphological variation in bermudagrass [52, 60]. In this study, a large proportion of the internode lengths in regions 8 and 10 were longer than those in plants in the vicinity, possibly due to the fact that the MAR in these areas was much higher than in the neighboring regions. This suggests that morphological variation in internode length in bermudagrass would arise when the variation in precipitation is abrupt. The higher AS (available sulfur) content of region of 5, which was second only to region of 13, had maximum values for PHT and RBH. This indicates that mid-longitude sites may be accompanied by optimal temperature and precipitation, which could allow soil characteristics to limit and or alter plant productivity.

Impacts of longitude on possible applications, and adaptive evolution of *C. dactylon* populationsThe tested traits were separated into three groups by cluster analysis; the dwarf-thin type, the middle-wide type, and the tall-stocky type. The bermudagrass plants in cluster A belong to the dwarf-thin type and can be used for lawns. The bermudagrass plants in cluster B have the best creeping features and are ideal for use in soil conservation and slope protection. The plants in cluster C have the best upright characters and belong to the tall-stocky type, which can be used for livestock forage. The bermudagrass plants in cluster C are mainly concentrated in higher longitude sites with less rainfall, and using this type of bermudagrass in breeding can contribute to conservation and reasonable utilization of precious water resources in arid environments.

The level of morphological variation in bermudagrass was found to be high, which enhances its ability to adapt to different environments along a longitudinal gradient. Compared with the other regions, the morphological traits were obviously different in marginal regions and central areas. The morphological trait sizes were generally larger in the marginal and central longitudes than in the other sampling sites along the longitudinal gradient. This result is supported by the non-linear trend in morphological trait values with respect to longitude, and the larger sizes of the morphological traits will occur in areas with intermediate and edge longitudes. In Lianyungang, which was highest longitude site of all the selected locations, the sizes of the morphological traits were generally larger than in the neighboring areas. This phenomenon can be explained by the observation that a high level of environmental variability, especially for rainfall, is present in Lianyungang, and it is thought that the highest level of morphological plasticity would occur in peripheral regions rather than in central areas, because environmental variation is probably the highest there [61, 62]. Earlier studies in other species have shown that higher levels of variability would be evident in marginal populations of *Acer platanooides* and *Picea abies* rather than in central populations [62, 63]. We found that two characters, width and diameter, showed a high degree of stability and were rarely affected by longitude and environmental factors, and the maximum values for width and diameter in bermudagrass were found in the middle longitude site Sanmenxia. Higher levels of diversity exist in central populations compared to marginal populations [64]. We can explain the results of our study from the following two perspectives. First, two populations of a species may have expanded their territory during their long-term evolutionary history, and these two populations then form a contact zone at some point in time. If the two populations were genetically differentiated, the species would thus have a higher diversity in the contact area [65]. Second, it has been suggested that high levels of environmental stress in edge sites could reduce morphological plasticity compared to that in central areas [66].

## Conclusions

The pronounced east-to-west longitudinal variations in morphological traits in bermudagrass show that longitude plays a major role in morphological variation. We observed that morphological trait sizes tended to increase with decreasing longitude in order for the plants to adapt to the changing environment, especially LLES, LWES, ILES, and ILS. However, the values for the morphological traits in plants from the edge and central sites were generally higher than in the other sampling locations along the longitudinal gradient because of the edge effects and the founder effects. Different combinations and interactions of environmental factors such as AS, exchangeable Ca, pH, MAT, and MAR at each site may obscure the general trends in trait changes in bermudagrass along the longitudinal gradient. Our research explored the evolutionary trends in morphological trait variation in wild bermudagrass populations along a longitudinal gradient, and provides abundant wild resources for breeding in bermudagrass. However, there are also shortcomings to this experiment; for example, we did not take into account that the content of soil nutrients could change with the changing of the seasons. Also, DNA molecular marker technology should be used to examine genetic variation in bermudagrass populations from China and other countries where *C. dactylon* grows wild.

## Data Availability

The datasets used and/or analyzed during the current study are available from the corresponding author on reasonable request.

## Abbreviations

AK: Available kalium
AN: Available nitrogen
AP: Available phosphate
AS: Available sulfur
CA: Cluster analysis
CV: Coefficient of variation
DES: Diameter of erect shoot
DS: Diameter of stolon
ILES: Internode length of erect shoot
ILS: Internode length of stolon
LLES: Leaf length of erect shoot
LLS: Leaf length of stolon
LWES: Leaf width of erect shoot
LWS: Leaf width of stolon
MAR: Mean annual rainfall
MAT: Mean annual temperature
P: Probability
PCA: Principal component analysis
PHT: Turf height
RBH: Reproductive branch height
SD: Standard deviation
SOM: Soil organic matter
TN: Total nitrogen
